# Supplementary material for: MORPHEUS Phase II–III Study: A Pre-Planned Interim Safety Analysis and Preliminary Results
Source: Cancers (Basel). 2022 Jul 28;14(15):3665. doi: 10.3390/cancers14153665 (PMC9367346; doi:10.3390/cancers14153665)

Supplemental Figure S1. Cumulative hazard of TME or death when comparing brachytherapy versus EBRT in the intention to treat population

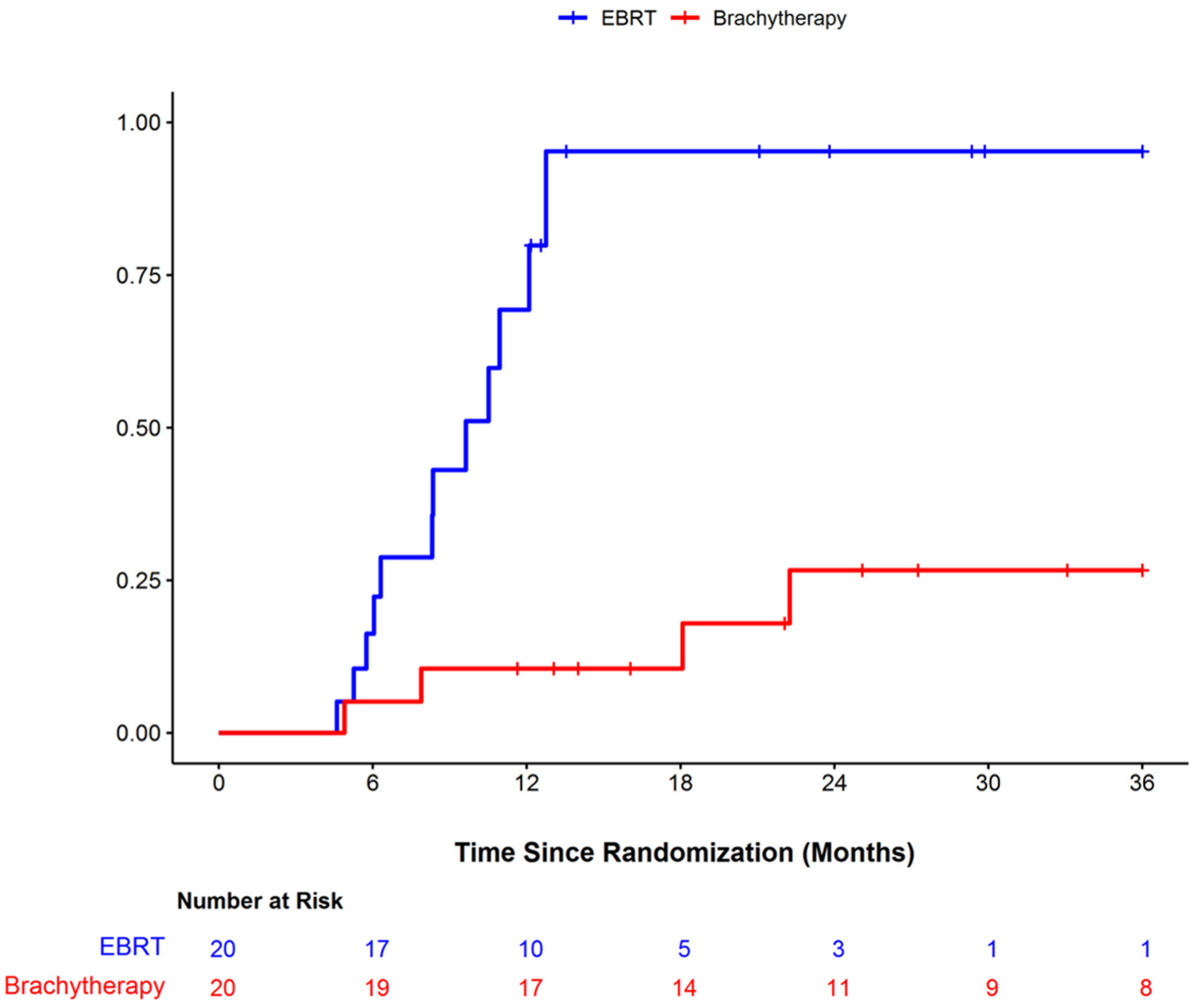

Supplement: Supplementary file 1 [file cancers-14-03665-s001.zip › cancers-1829353-Supplemental Figure S1.pdf]
